# Supplementary material for: Infigratinib (BGJ 398), a Pan-FGFR Inhibitor, Targets P-Glycoprotein and Increases Chemotherapeutic-Induced Mortality of Multidrug-Resistant Tumor Cells
Source: Biomedicines. 2022 Mar 3;10(3):601. doi: 10.3390/biomedicines10030601 (PMC8945560; doi:10.3390/biomedicines10030601)
Supplement: Supplementary file 1 [file biomedicines-10-00601-s001.zip › biomedicines-1613491-supplementary.pdf]

A.

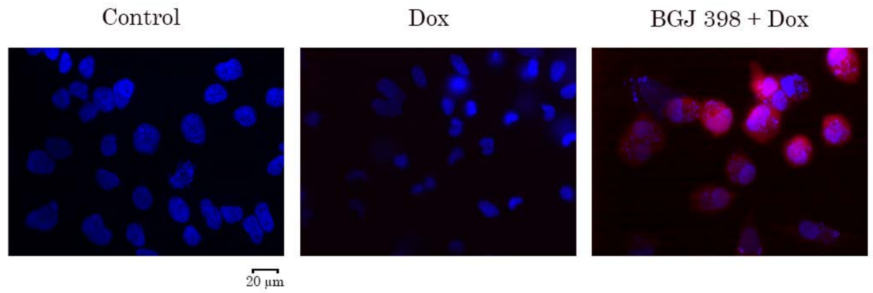

B.

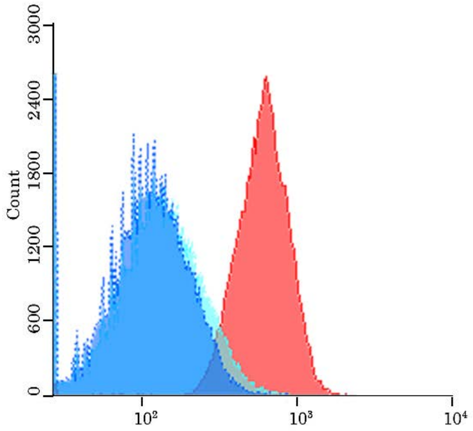

Supplementary Figure S1. Fluorescence microscopy **(A)** and FACs **(B)** analysis of the intracellular accumulation of doxorubicin (DOX) in Tx-R GIST T-1 cells. The cells were first incubated with BGJ 398 (1 μM) (*right*) or DMSO as a control (*middle*) for 1 h and then incubated with 40 μM DOX for an additional 1.5 h. After the wash-out with a pre-warmed culture medium, the BGJ 398-treated cells were additionally incubated with BGJ 398 for 1 h and analyzed by FACs or fluorescence microscopy. For FACs analysis (**B**) histogram illustrating DMSO-treated cells is shown in blue color, cells treated with Dox (40 μM) alone (*deep blue*) or in combination with BGJ 398 (1 μM) (*red*).

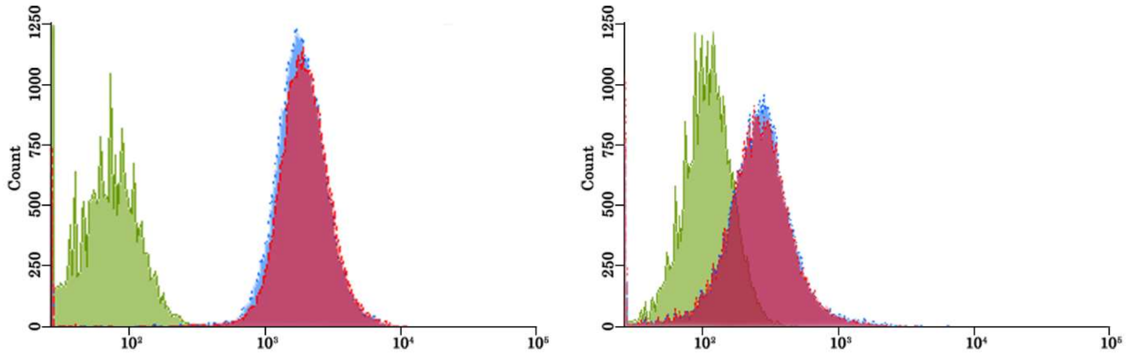

Supplementary Figure S2. The intracellular accumulation of Dox in drug-sensitive parental HCC 1806 cells (*left pane*l) and Tx-R HCC 1806 cells (*right panel*). The cells were treated with DMSO (*green*), Dox (45 μM) alone (*blue*) or in combination with PD 173074 (100 μM) (*brown*). The fluorescence intensity was analyzed by FACs. Representative histograms of at least three independent experiments are shown.

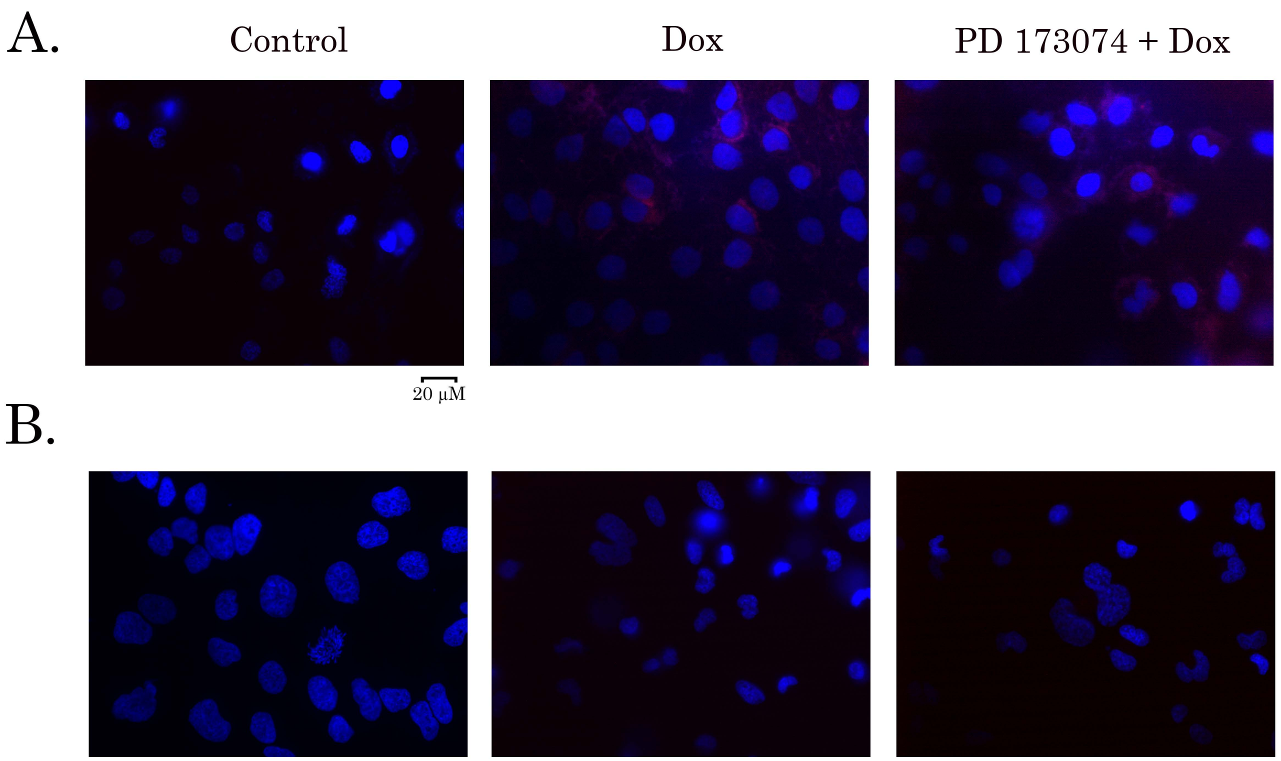

Supplementary Figure S3. Fluorescence microscopy analysis of the intracellular accumulation of doxorubicin (DOX) in Tx-R HCC 1806 breast cancer **(A)** GIST T-1 **(B)** cells. The cells were first incubated with PD 173074 (100 μM) (*right*) or DMSO as a control (*middle*) for 1 h and then incubated with 40 μM DOX for an additional 1.5 h. After the wash-out with a pre-warmed culture medium, the PD 173074-treated cells were additionally incubated with FGFR inhibitor for 1 h. The non-fixed slides were counterstained with Hoechst 33342 for 1 min to outline the nuclei and processed for fluorescence microscopy to obtain the merged images.

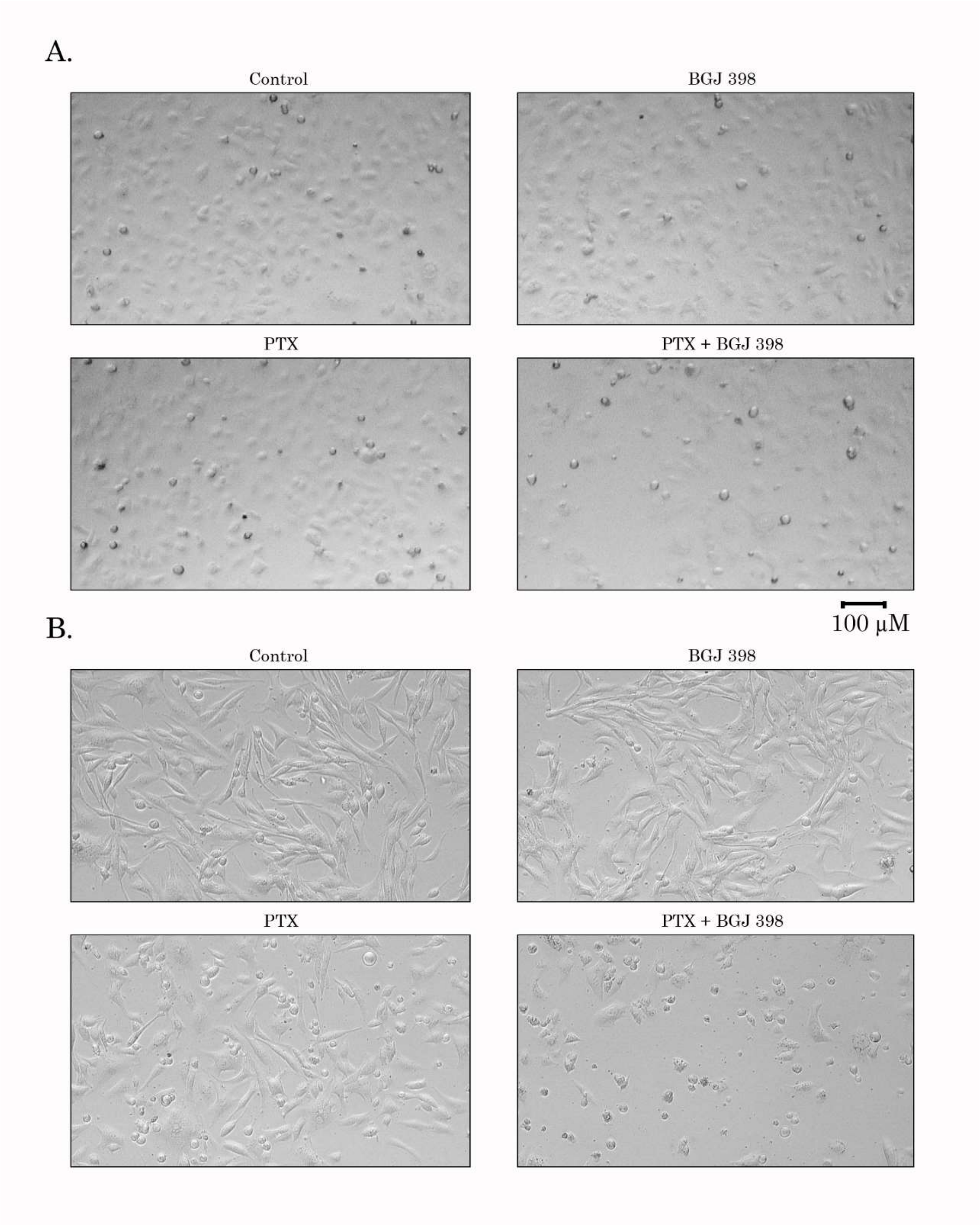

Supplementary Figure S4. Morphological changes in Tx-R HCC 1806 breast cancer cells **(A)** and GIST T-1 cells **(B)** after treatment with DMSO (negative control), PTX, BGJ 398 alone or with combination of PTX and BGJ 398 for 48 h. Cells were subjected to light microscopy (Leica). Magnification 10×, scale bars 100 μM.

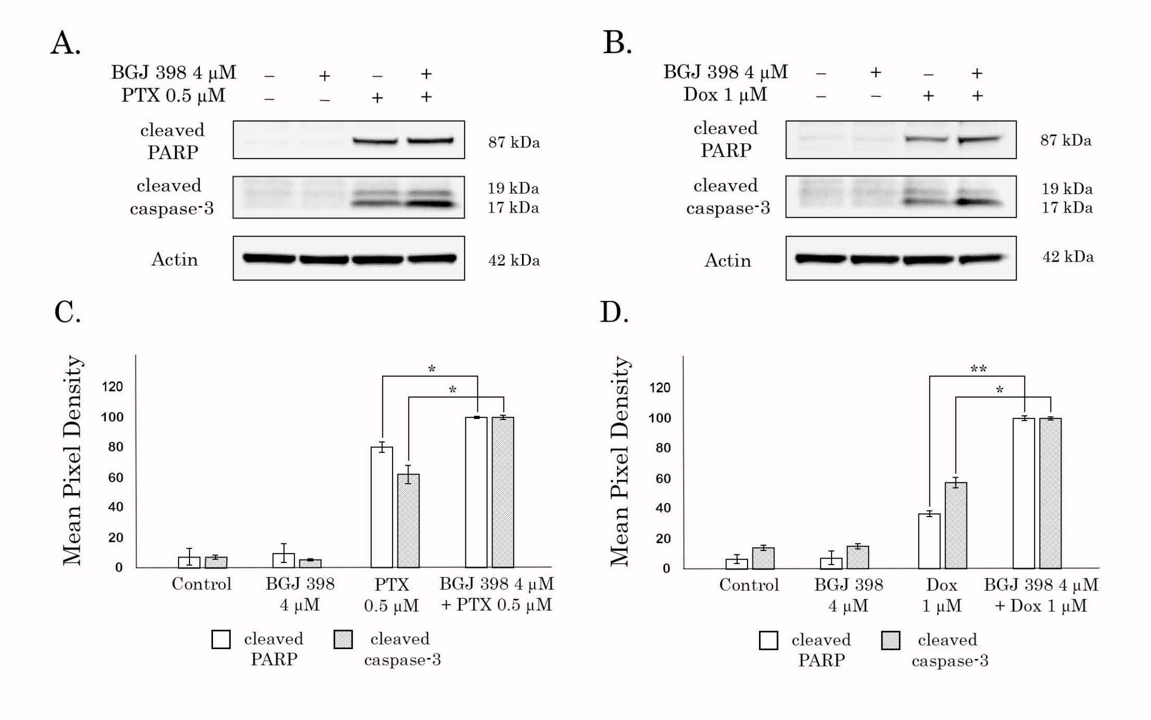

Supplementary Figure S5. BGJ 398 potentiates apoptosis of Tx-R GIST T-1 cells treated with combination of PTX (or Dox). Immunoblot analysis for apoptosis markers (cleaved forms of PARP and caspase-3) in Tx-R GIST T-1 cancer cells after treatment with DMSO (negative control), PTX, BGJ 398 alone or in combination (**A**) and DMSO (negative control), Dox, BGJ 398 alone or in combination (**B**) for 48 h. Actin stain is used as a loading control. (**C-D**) Quantification by mean pixel density in the cleaved forms of PARP and caspase-3 in Tx-R GIST T-1 cells treated with DMSO (negative control), PTX, BGJ 398 alone or in combination (**C**) and DMSO (negative control), Dox, BGJ 398 alone or in combination (**D**). Values are means ± SD, N = 3. * $p < 0.05$, ** $p < 0.01$ versus untreated cells.

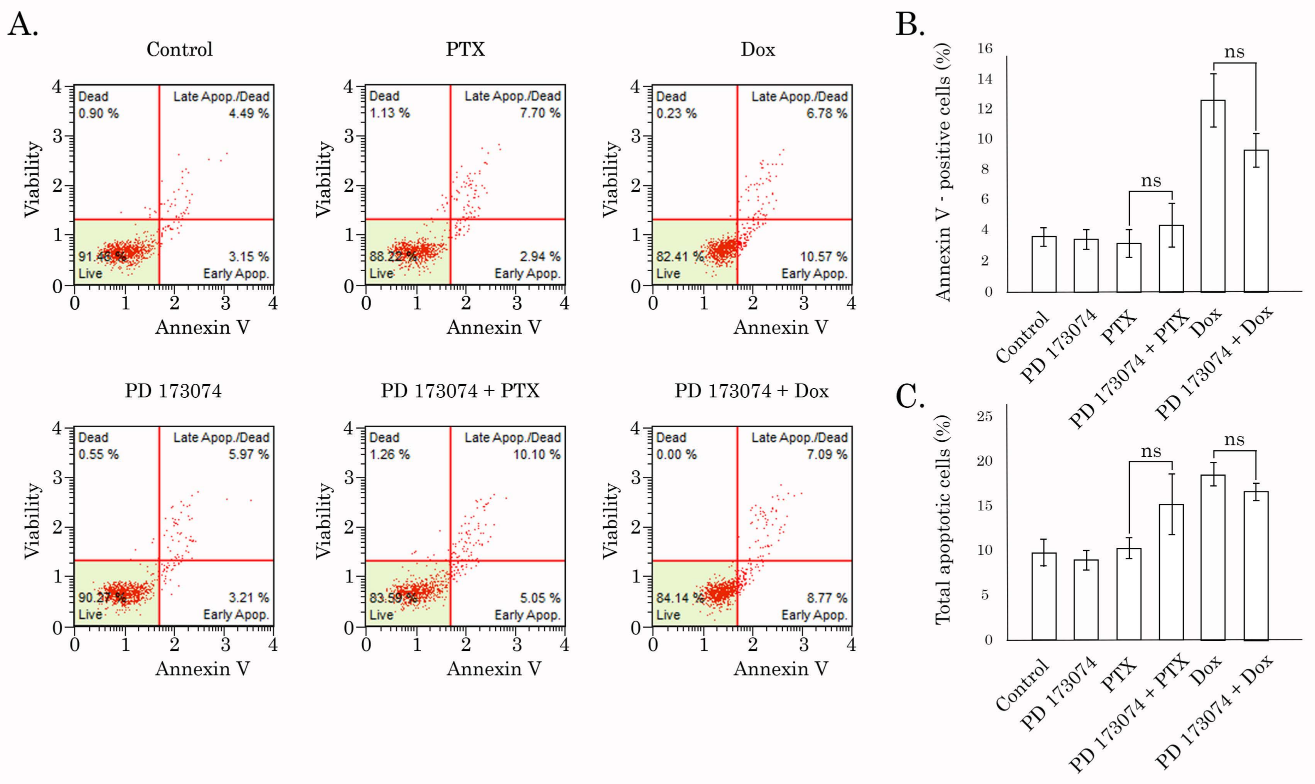

Supplementary Figure S6. FACs analysis for apoptotic markers in Tx-R HCC 1806 breast cancer cells treated with PD 173074 and PTX (or Dox) alone or in combination. **(A)** Representative histograms illustrating an increase of early (Annexin V-positive/propidium iodide (PI)-negative) and late (double-positive) apoptotic cells in the HCC1806 Tx-R TNBC cancer cell line treated with DMSO (negative control), PTX (1μM), Dox (0.5 μM), PD 173074 (100 μM) alone or in combination for 48 h. **(B)** Quantitative analysis of the early apoptotic cells after the treatment as indicated above. **(C)** Quantitative analysis of the total apoptotic cells after the treatment as indicated above. ns - no statistically significant difference.

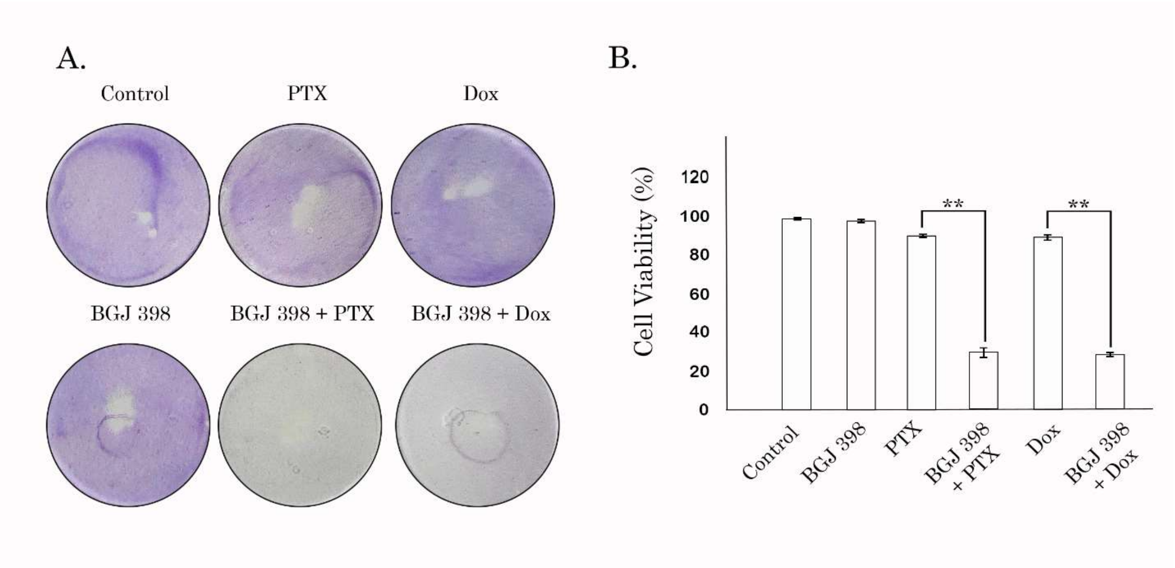

Supplementary Figure S7. The impact of BGJ 398 used in combination with PTX or Dox on proliferation and survival of Tx-R GIST T-1 cells. **(A)** Crystal violet staining of Tx-R GIST T-1 cells that were treated with BGJ 398 alone or in combination with PTX (or Dox) for 96 h. The cells treated with DMSO were used as a control. The cells treated with PTX or Dox alone illustrated chemoresistance of Tx-R GIST T-1 cells. The culture dishes were fixed with ice-cold 100% methanol, stained with crystal violet, and photographed; **(B)** Quantification of crystal violet staining of Tx-R GIST T-1 cells treated with PTX and Dox alone or in combination with BGJ 398. The plates were dried, crystal violet was dissolved using 0.1% SDS solution, and absorbance was measured at 590 nm. The graphs represent the mean ± SD, N=4. ** $p < 0.01$.

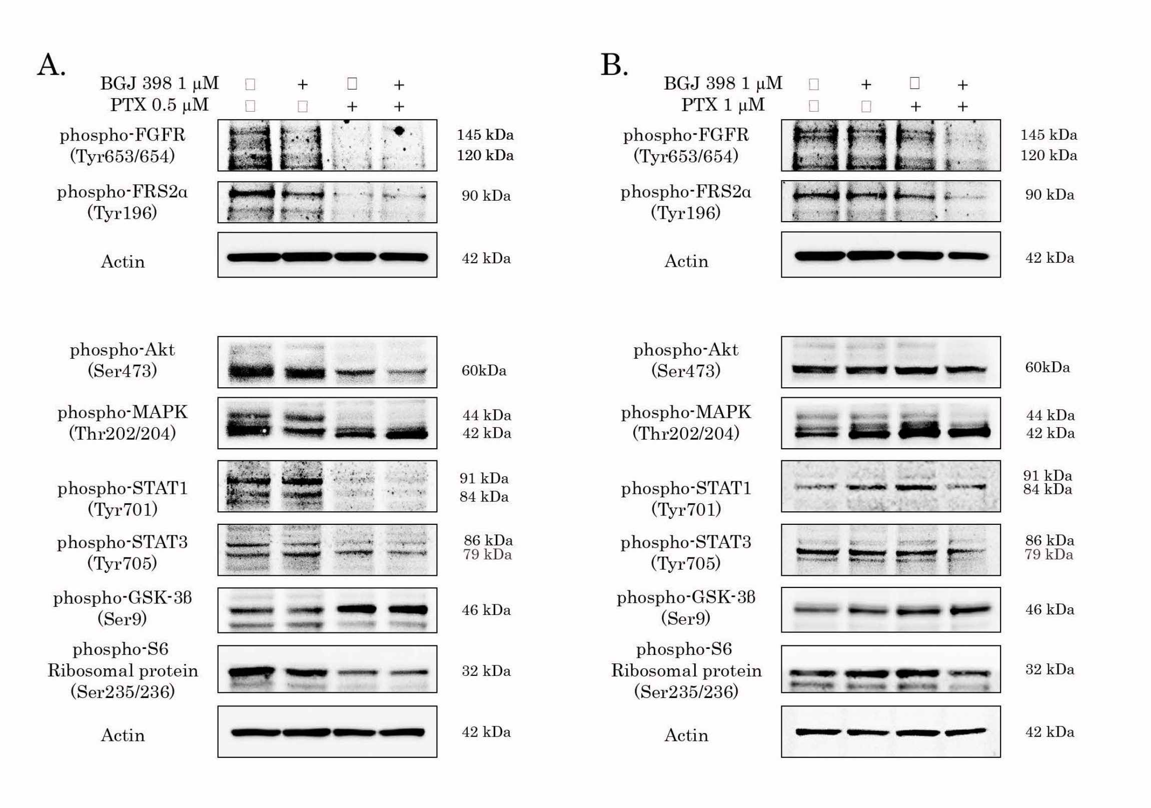

Supplementary Figure S8. Expression of the markers of FGFR-signaling in naive **(A)** and Tx-R **(B)** HCC 1806 cells treated with DMSO (control), BGJ 398 and PTX alone or in combination for 72h.

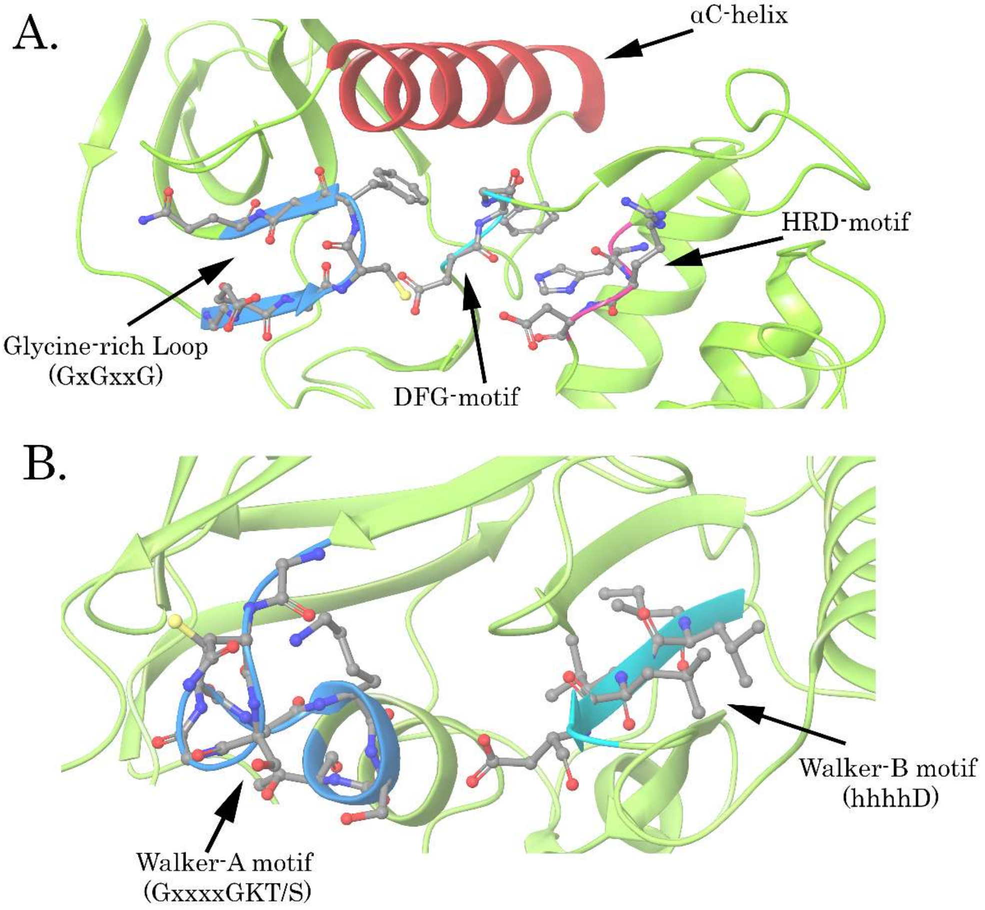

Supplementary Figure S9. 3D structure comparison of ATP-binding sites of FGFR2 **(A)** and ABCB1 **(B).** Arrows indicate the most important amino acid motifs of the two domains. The amino acid sequence of motifs is written as a IUPAC one-letter code. x means any amino acid, h means any hydrophobic amino acid.

Supplementary Table S1. Fold-increase of ABC-transporters mRNA levels in Tx-R cancer cells compared with the parental cells (determined by RT-qPCR). Values are averages of 3 experiments.

	Genes	*MDR1*	*MRP1*	*MRP2*	*MRP3*	*MRP4*	*MRP5*	*MRP7*
HCC 1806 Tx-R	Fold change	10,56	12,95	8,17	2,81	2,21	33,82	3,81
GIST T-1 Tx-R		1,71	0,72	-	1,06	0,10	1,89	1,89

Supplementary Table S2. Fold-decrease of ABC-transporters mRNA levels in BGJ 398-treated Tx-R cancer cells compared with non-treated Tx-R cells (determined by RT-qPCR). Values are averages of 3 experiments.

	Genes	*MDR1*	*MRP1*	*MRP2*	*MRP3*	*MRP4*	*MRP5*	*MRP7*
HCC 1806 Tx-R	Fold change	0,29	0,96	0,36	0,01	0,41	0,07	0,22
GIST T-1 Tx-R		0,49	0,24	-	0,40	12,82	0,70	0,69
